# SCG2: A Prognostic Marker That Pinpoints Chemotherapy and Immunotherapy in Colorectal Cancer

**DOI:** 10.3389/fimmu.2022.873871

**Published:** 2022-07-01

**Authors:** Siyuan Weng, Zaoqu Liu, Xiaofeng Ren, Hui Xu, Xiaoyong Ge, Yuqing Ren, Yuyuan Zhang, Qin Dang, Long Liu, Chunguang Guo, Richard Beatson, Jinhai Deng, Xinwei Han

**Affiliations:** ^1^ Department of Interventional Radiology, The First Affiliated Hospital of Zhengzhou University, Zhengzhou, China; ^2^ Interventional Institute of Zhengzhou University, Zhengzhou, China; ^3^ Interventional Treatment and Clinical Research Center of Henan Province, Zhengzhou, China; ^4^ Faculty of Engineering and Information Technology University of Technology Sydney, Sydney, NSW, Australia; ^5^ Department of Respiratory and Critical Care Medicine, The First Affiliated Hospital of Zhengzhou University, Zhengzhou, China; ^6^ Department of Colorectal Surgery, The First Affiliated Hospital of Zhengzhou University, Zhengzhou, China; ^7^ Department of Hepatobiliary and Pancreatic Surgery, The First Affiliated Hospital of Zhengzhou University, Zhengzhou, China; ^8^ Department of Endovascular Surgery, The First Affiliated Hospital of Zhengzhou University, Zhengzhou, China; ^9^ King’s College London, School of Cancer and Pharmaceutical Sciences, Guy’s Cancer Centre, London, United Kingdom; ^10^ Richard Dimbleby Laboratory of Cancer Research, School of Cancer and Pharmaceutical Sciences, King’s College London, London, United Kingdom

**Keywords:** colorectal cancer, chemotherapy, prognosis, immunotherapy, biomarker

## Abstract

**Background:**

Fluorouracil (FU)-based chemotherapy regimens are indispensable in the comprehensive treatment of colorectal cancer (CRC). However, the heterogeneity of treated individuals and the severe adverse effects of chemotherapy results in limited overall benefit.

**Methods:**

Firstly, Weighted gene co-expression network analysis (WGCNA) identified modules tightly associated with chemotherapy response. Then, the in-house cohort and prognostic cohorts from TCGA and GEO were subjected to Cox proportional hazards model and survival analysis to ascertain the predictable function of SCG2 on the prognosis of CRC patients. Finally, we performed *In vitro* experiments, functional analysis, somatic mutation, and copy number variation research to explore the biological characteristics of SCG2.

**Results:**

We identified red and green as the modules most associated with chemotherapy response, in which SCG2 was considered a risky factor with higher expression predicting poorer prognosis. SCG2 expression in the APC non-mutation group was remarkably higher than in the mutation group. The mutation frequencies of amplified genes differed significantly between different SCG2 expression subgroups. Besides, CRC cell lines with SCG2 knockdown have reduced invasive, proliferative, and proliferative capacity. We discovered that the SCG2 high expression subgroup was the immune hot type and considered more suitable for immunotherapy.

**Conclusion:**

This study demonstrates the clinical significance and biological characteristics of SCG2, which could serve as a promising biomarker to identify patients who may benefit from chemotherapy and immunotherapy.

## Introduction

Colorectal cancer (CRC) accounts for approximately 10% of all annual diagnosed cancer and oncology-related deaths worldwide and is the second leading contributor of cancer death worldwide (1). Although the development of early screening methods and efficacious treatment options has contributed to a dramatic improvement in colorectal cancer patients, CRC remains a considerable health burden (2–4). Since the 90s of the 20th century, fluorouracil (FU)-based adjuvant chemotherapy has been an essential choice to decrease the risk of advanced colorectal cancer (5). For stage II patients with risk factors, fluoropyrimidine monotherapy is often used. In resected stage III colorectal cancer, adjuvant fluoropyrimidine alone reduces the risk of death by 10% to 15%, with an additional 4% to 6% diminution in the risk of death when treatment consists of oxaliplatin-based combination therapy (6). Although adjuvant chemotherapy has achieved remarkable results, the heterogeneity of the tumor and risk factors of disease make the clinical outcome and treatment response of CRC patients very different (7). However, there is considerable potential to improve the benefit ratio by adopting a more personalized approach. The noninferiority of 3-month adjuvant chemotherapy illustrates this possibility compared to 6-month standard treatment duration in patients with low-risk stage III and high-risk stage II CRC (8, 9).

The tremendous benefit achieved in the past several years with the appearance of immunotherapy and checkpoint inhibitors (ICIs) has revolutionized the field of oncology, particularly regarding the therapy of solid tumors (10). Immuno-checkpoint regimens received FDA approval in 2017 for CRC patients with defective mismatch repair (dMMR) or high-level microsatellite instability (MSI-H). In addition, more meticulous tumor biological stratification has led to successfully durable responses to PD1 and CTLA4 inhibitors in some patients, such as tumor mutation burden (TMB) and tumor environment (TME) (11). By contrast, ICIs perform tiny efficiency on tumors with mismatch repair proficient (pMMR), microsatellite-stable (MSS), or low levels of microsatellite instability (MSI-L), which account for a large proportion of CRC (12). Besides, TMB is also not the only biological marker to judge the response to immunotherapy (13). Therefore, based on the polymorphisms of tumor biology, it is imperative to investigate novelty biomarkers that can promote immunotherapy precisely in CRC.

While chemotherapy and immunotherapy exert their unique advantages in the multimodality treatment of CRC, the limitations of monotherapy with each of them make the therapeutic benefit applicable to only a minority of CRC patients. A growing body of research has shown that chemotherapy is simple tumor suppression and involves positive immune system regulation. CRC treatment with conventional chemotherapeutic agents also represents a monstrous burden for the patient’s apparatus due to the high toxicity and the correspondingly low response (14). Recent advancements in deciphering the biology and drivers of early-stage disease and the microenvironment promise to translate into patient-specific therapeutic strategies (6). Stratified approach or biomarkers to guide precise treatment of CRC will prevent unnecessary burdens on patients both physically, mentally, and financially. However, there is no proposed biomarker to determine the prognosis while also predicting the efficacy of chemotherapy and immunotherapy in patients. In the present research, we integrated the chemotherapy and prognosis data analysis by bioinformatics methods to identify a marker that predicted prognosis and response to chemotherapy and discovered that the biomarker also predicted potential immunotherapeutic responding.

## Methods

### Public Data Access and Proceed

Somatic mutation profiles, copy number alteration (CNA), RNA sequencing data, and correspondent clinical information of CRC sourced from The Cancer Genome Atlas (TCGA) portal. Expression microarrays datasets containing chemotherapy (GSE19860, GSE62080, GSE69657) and survival (GSE161158, GSE17536, GSE17537, GSE29621, GSE38832, GSE39582, GSE87211) cohorts were accessed from the Gene Expression Omnibus (GEO) database. Meta-GEO cohort consisted of 3 chemotherapy databases, in which raw data were acquired and further handled *via* a robust multi-array averaging algorithm (RMA) incorporated in the “affy” R package (15). We rectified batch effects using the ComBat function in the “sva” R package.

### WGCNA and Identifying Key Module

Weighted Gene Co-expression Network Analysis (WGCNA) facilitates network-based gene screening, detecting markers with specific characteristics, such as treatment response. To define latent highly co-expressed clusters of genes, we developed the gene profiling of the Meta-GEO cohort into gene co-expression networks using the “WGCNA” package (16, 17). The clustering analysis of samples was achieved *via* the “hclust” function to validate and remove outliers. An appropriately elected soft power threshold that could accentuate robust associations of genes and penalize low associations ensures the scale-free network. The adjacency matrix is founded by analyzing the Pearson correlation between each extracted gene pair and transformed into a topological overlap matrix (TOM) and a corresponding dissimilarity (1-TOM). The “DynamicTreeCut” algorithm implemented network configuration and consensus module detection. The module eigengene (ME) was calculated for each *module*, representing the gene expression profiles of a given module. The modules with the high correlation coefficient between ME profiles and clinical feature information were considered candidate modules and selected for consequent analysis.

### Functional Enrichment Analysis

Gene ontology (GO) and Kyoto Encyclopedia of Genes and Genomes (KEGG) combine genomic information with advanced functional information to explain the function of genes. The enrichment of GO compasses biological process (BP), cellular component (CC), and molecular function (MF). We conducted the GO term and KEGG pathway analysis with the “clusterProfiler” R package for genes based on feature-related modules. P-value <0.05 was deemed to be statistically significant for functional annotation.

### Identification and Validation of Prognostic Hub Genes

To further identify hub genes that tightly connect with chemotherapy response, we adopted the ‘pROC’ R package to calculate the area under the receiver operating characteristic curve (AUC) to evaluate the chemotherapy-predicted power of the candidate module genes. Gene, whose AUC >0.7 simultaneously in 3 chemotherapy cohorts, was selected to perform the univariate Cox analysis to determine prognostic genes in relapse-free and overall survival cohorts. Kaplan-Meier survival curves with log-rank tests validated the foreboding power of these genes (P <0.05). Subsequently, the results obtained by the external queue by the above method are further elaborated in the in-house chemotherapy cohort.

### Validation of Gene Expression by qRT-PCR

The genes associated with both prognosis and chemotherapy were detected in quantitative real-time PCR analysis. The clinicopathological features of each patient are summarized in **Table S1**. Total RNA was extracted from the human tumor and adjacent normal tissues using TRIzol reagent (Invitrogen, Carlsbad, CA, USA) and reverse transcript using TIANScript RT kit (Servicebio, Wuhan, China). The expression value of the target genes was normalized to GAPDH and then log2 transformation for subsequent analysis. The primer sequences of the included four genes and GAPDH are shown in **Table S2**. The 2-ΔΔCT approach was applied to compute the relative RNA expression of each gene.

### Immunohistochemistry

Immunohistochemistry (IHC) was performed using an anti-SCG2 (BS-1988R, 1:500) antibody. Staining percentage scores were classified as follows: 1 (1%-25%), 2 (26%-50%), 3 (51%-75%), and 4 (76%-100%), and staining intensity was scored 0 (signal less color) to 3 (light yellow, brown and dark brown). The stained tissues were scored by three individuals blinded to the clinical parameters, and the IHC scores were determined by percentage and intensity scores.

### Gene Set Enrichment Analysis

For confirming the biological features of SCG2, we counted the correlations with the other genes and ordered genes based on results. The ordered gene list was input GSEA analysis to investigate whether strongly correlated genes gathered in meaningfully functional pathways. The annotated gene set chosen as the reference gene set included c5.go.v7.4.symbols.gmt and c2.cp.kegg.v7.4.symbols.gmt. False discovery rate (FDR) <0.05 and p-value <0.01 were regarded significant as described previously.

### Gene Set Variation Analysis

For further investigating the differences of SCG2 about biological processes, we divided the samples from TCGA datasets into two groups depending on the median expression of SCG2 and subsequently utilized GSVA enrichment analysis to investigate whether differentially expressed genes in the two groups gathered meaningfully functional pathways. The annotated gene set hallmarks downloaded from the GSEA portal were chosen as the reference gene set. The absolute values of t >1 were considered significant as described previously (18).

### The Landscape of Somatic Mutation

Tumor mutation burden (TMB) was considered the biomarker representing macroscopic alterations of genomic mutations. We calculated all base substitutions and insertions or deletions in the coding regions of the target genes. The top 30 genes in mutation frequency were defined as the driver genes by the ‘maftool’ R package. In accord with the expression of SCG2, the mutation landscape of the driver genes in two groups of samples was described respectively with regarding p-value <0.05 as remarked differences. Univariate and multivariate logistic regression analysis investigated the dependence of driver gene mutations on SCG2 with additional clinical information, including TMB, age, gender, and stage.

### Copy Number Alteration Analysis

We conducted the* *GISTIC* *2.0 pipeline to determine the significantly amplified and deleted genome regions. A mutational landscape map of CNA was constructed by amplifying and deleting each of the top 15 genes in copy number. We compared the expression differences of each gene in groups with different SCG2 expressions and implemented logistic regression analysis to explore the role of SCG2 in CNA. Univariate logistics used SCG2 expression as an independent variable. In multivariate logistics regression, SCG2 expression and fraction of genome gained (FGG) were included as independent variables when amplified genes were dependent variables, while SCG2 expression and fraction of genome lost (FGL) were included as independent variables when deleted genes were dependent variables.

### Cell Transient Transfection, RNA Extraction and qRT-PCR

The current research used two cell lines comprising human CRC cell lines, HCT116 and SW480. CAL-27 and CAL-33 were incubated in DMEM (Solarbio, Beijing, China) containing 10% fetal bovine serum (Bioind, Kibbutz Beit Haemek, Israel), preserved in the humidified incubator with 5% CO_2_, 37°C. RiboFECT™ CP (RiboBio, Guangzhou, China) was used to transfect Negative Control (NC) and SCG2 siRNAs (RiboBio, Guangzhou, China) into CRC cells according to the manufacturer’s instruction. The plates were placed in the incubator for 48 hours, and total RNA was extracted for qRT-PCR. The siRNA sequences were as follows:

siRNA#1 5’ CCTATGCCTTGAATTCAGA dTdT 3’,
siRNA#2 5’ GCCGAATGGATCAGTGGAA dTdT 3’,
siRNA#3 5’ CCAAGTGAAGCGAGTTCCT dTdT 3’.

### Wound Healing Assay

The constructed NC and SCG2 siRNA cells were seeded in 24-well culture plates (1 × 10^5^/well) and placed at 37°C, Incubated overnight in a 5% CO_2_ incubator. Remove the medium and use a 10 μl pipette tip to scratch and mark the surface of the inoculated cells. Wash gently with PBS three times. Photograph the scratches at 0 h and 48 h. The experiment is repeated three times. Measure the distance of cell migration to the injured area during this period.

### Transwell Assay

Transwell chambers were utilized to measure the invasion and migration ability of the cells. Approximately 4 × 10^4^ transiently transfected cells were cultured in the upper chamber with serum-free medium, while a complete medium was added to the lower chamber. Cells were maintained at 37°C for 24 hours, washed with physiological saline, and fixed with methanol. After that, 0.1% crystal violet stain solution (Solarbio) was used to stain the cells. Finally, cells were photographed under a microscope, and stained cells were counted.

### Colony Formation Assay

Each cell line was inoculated in a gradient of 50, 100, and 200 cells per dish, respectively, and cells were cultured in a 5% CO_2_ incubator at 37°C for 2 weeks. At the end-point, the cells were washed with cold phosphate-buffered saline (PBS) twice, fixed with 4% paraformaldehyde for 15 minutes, and stained with 1% crystal violet solution for 20 minutes at room temperature. The visible colony numbers were counted. This experiment was performed in triplicate. Finally, to calculate the clone formation rate (Clone formation rate =(number of clones/number of inoculated cells) × 100%).

### Cell Counting Kit-8 Assay

Approximately 1 × 10^4^ transiently transfected cells in 100 μL medium were maintained in 96-well plates. After 24, 48, 72, and 96 hours incubation, 10 μL cell counting kit-8 assay (CCK8) solution was added to each well. After 2 hours of additional incubation, the optical density (OD) value at 450 nm was measured with a microplate reader.

### 5-Ethynyl-2’-Deoxyuridine Assay

First, HCT116 and SW480 cells were grown in a 5-ethynyl-2’-deoxyuridine (EdU) solution for 2 hr. The cells were fixed with PBS containing 4% paraformaldehyde. Finally, the fixed cells were deposited in 70% ethanol, and then Cell-Light™ EdU Apollo^®^567 *In Vitro* Imaging Kit (RiboBio, China) was used to dye cells. Cell growth was observed by fluorescence microscopy.

### Evaluation of Immune Infiltration and Immunotherapy Response

Single-sample gene set enrichment analysis (ssGSEA) quantified the infiltration level of immunity cells in individual cancer samples based on the ‘ssGSEA’ R package. The deconvolution method was employed in this research covering 28 immune cells in innate immunity (19). Then, we predicted clinical reactions to ICIs according to pre-treatment expression data of tumors with the Tumor Immune Dysfunction and Exclusion (TIDE) web tool (http://tide.dfci.harvard.edu/). The TIDE framework evaluates immune evasion by integrating T cell dysfunction and rejection (20). By the TIDE tool, we acquired the results of the bioinformatics evaluation of each patient’s exposure to immunotherapy. The Subclass Mapping (SubMap) is an unsupervised clustering algorithm discovering shared subtypes among separate queues. With the SubMap approach, we analyzed similar transcriptome expression patterns between SCG2 differentially expressed groups and patients with distinct immunotherapy responses (21). FDR <0.05 suggested that the two subcategories were significantly similar.

### The Prediction of Potential Drug

We employed the Connectivity Map (CMap), a data-driven, systematic approach for discovering associations among genes, chemicals, and biological conditions, to search for candidate compounds that might target pathways associated with CRC (22). To further investigate the mechanism of actions (MoA) and drug target, we performed specific analysis through CMap tools.

### Statistical Analysis

R version 4.1.0 was employed to conduct all statistical analyses and plotting while processing data. The correlation of gene expression with gene significance for drug response was evaluated using the Person or Spearman correlation coefficient. The Wilcoxon rank-sum test and Kruskal-Wallis test were applied to test for differences between two and multiple groups. The Kaplan-Meier analysis was performed *via* the “survival” R package, and the log-rank test was applied to compare the survival differences among three phenotypes. All p-values were bilateral, and less than 0.05 were considered statistically significant.

## Results

### Modules Relevant to Chemotherapy Response

**Figure 1:**
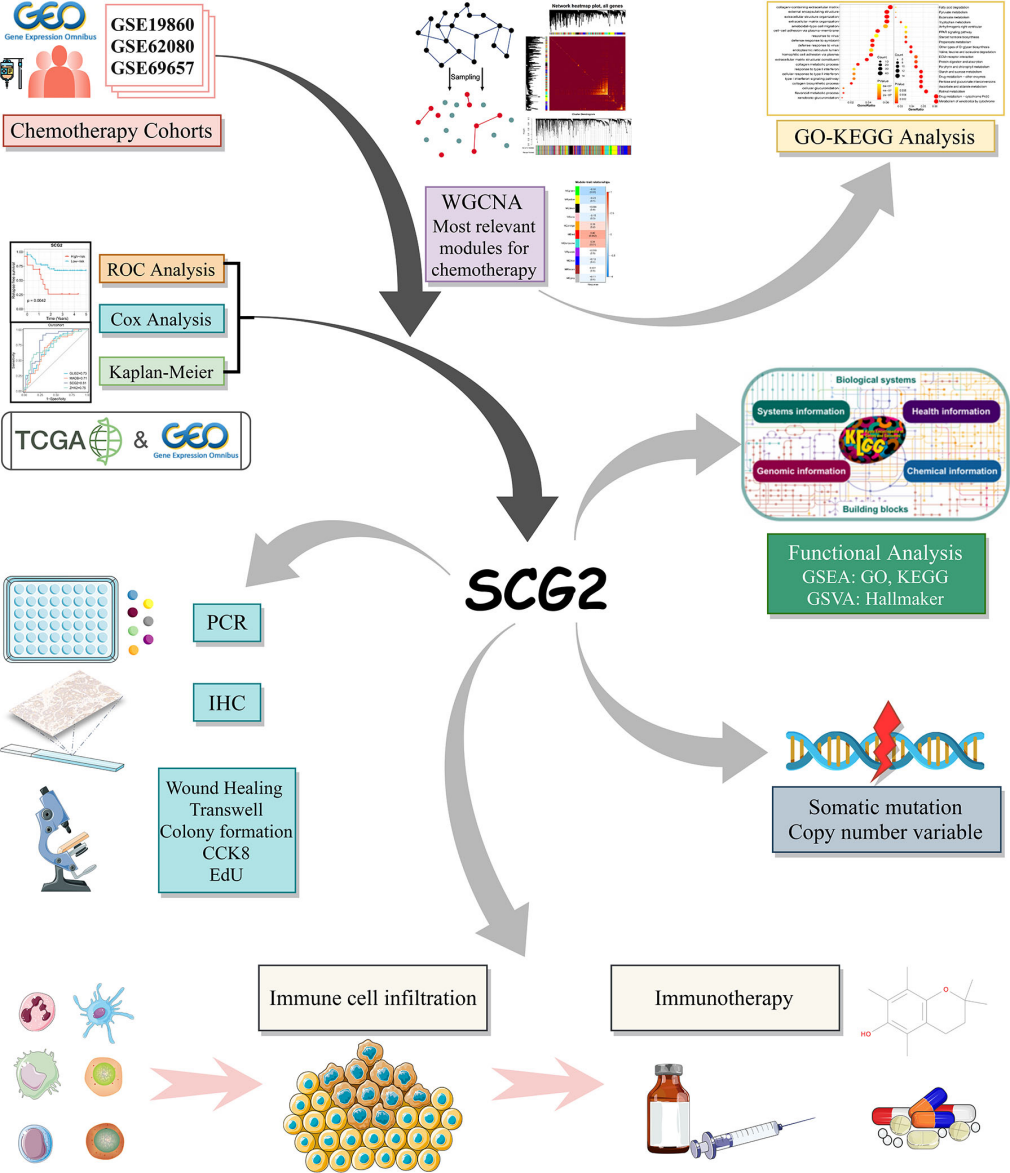
The flow chart of this study.

**Figure 2:**
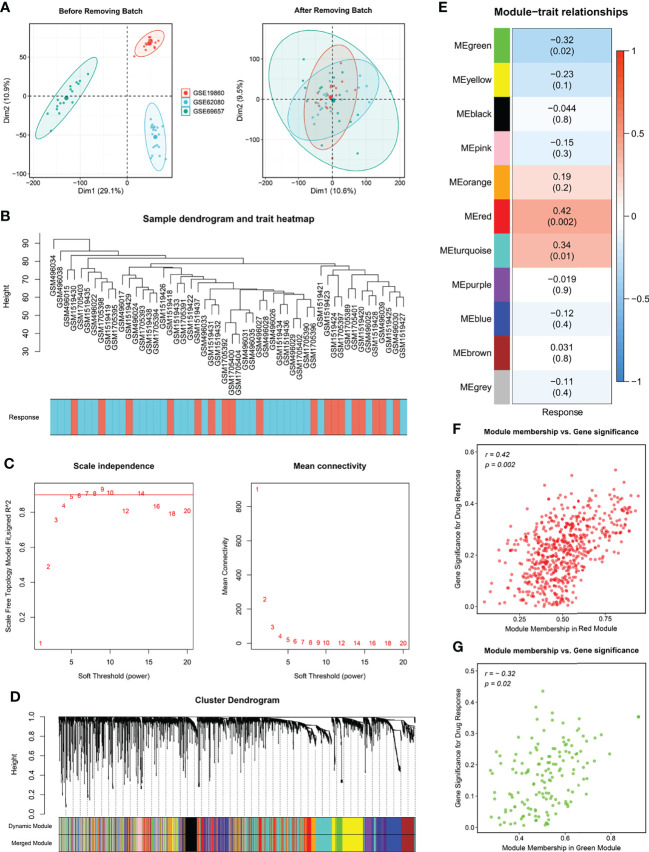
Modules relevant to chemotherapy response. **(A)** Raw data from GSE19860, GSE62080 and GSE69657 were batch corrected to form a Meta cohort. **(B)** Remaining samples after discarding outliers. **(C)** Scale-free topological indices at various soft-thresholding powers. **(D)** Gene clustering diagram based on hierarchical clustering under optimal soft-thresholding power. **(E)** Correlations between gene modules and chemotherapy response. **(F, G)** The correlation between the key modules (red, **F**; green, **G**) memberships and the gene significance for chemotherapy response.

The flow chart of this study is shown in **Figure 1**. For identifying significant modules associated with the response of Fluoropyrimidine-based chemotherapy in CRC, we removed outlying samples from the Meta-GEO cohort after batch correction (**Figures 2A, B**) and performed WGCNA. The optimal β =6 considered the soft threshold ensured that the constructed networks were scale-free (scale-free R2>0.90, **Figure 2C**). To make the segmentation of modules easier, we transformed the adjacency matrix to the topological overlap matrix (TOM), which is displayed in **Figure S1A**. Then, using a cutoff of 0.25 and a minimum module size of 50 contributed to 18 modules (**Figure 2D**). An eigengene adjacency heatmap depicted the correlations between modules (**Figure S1B**). Subsequently, we used a heatmap to explore the relationship between modules and chemotherapy response (**Figure 2E**). The red block (r =0.42, *P* =0.002) and the green block (r =-0.32, *P* =0.02) had the greatest correlations with chemotherapy response. The expression level of 623 genes in the red block was positively correlated with chemotherapy response, while 170 genes in the green block were negatively correlated with chemotherapy response (**Figures 2F, G**). It implied that 793 genes serve more critical functions in the molecular mechanisms of chemotherapy response.

### Functional Analysis and Identify Prognostic Genes in Modules

**Figure 3:**
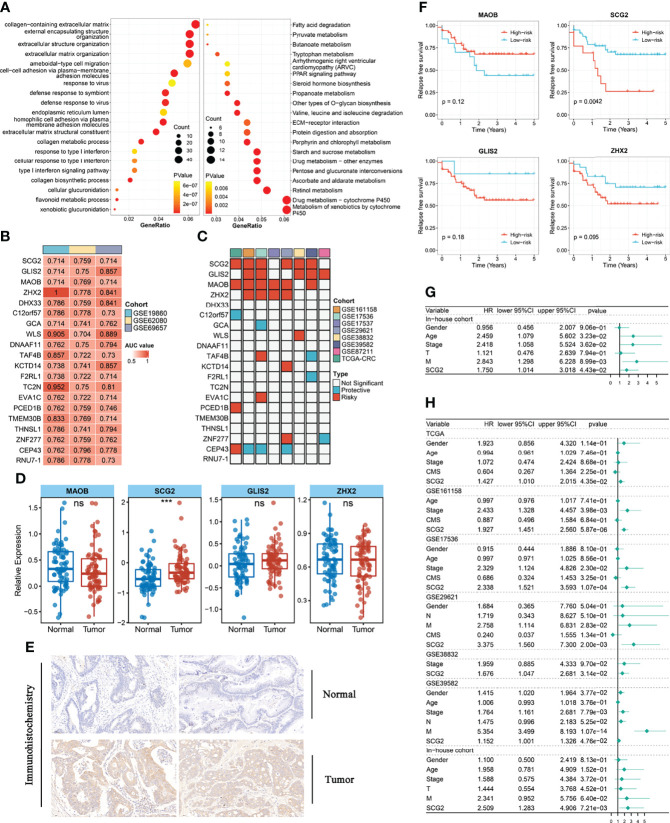
Functional analysis and identify prognostic genes in modules. **(A)** Gene ontology (GO) and Kyoto Encyclopedia of Genes and Genomes (KEGG) enrichment analysis of genes in key modules. **(B)** The 20 module genes with AUC values >0.7 in all chemotherapy cohorts. **(C)** Univariate analysis with relapse-free survival (RFS) as the outcome event and the expression of the above 20 genes as independent variables. **(D)** Different expression of GILS2, MAOB, SCG2, ZHX2 between tumor and normal tissues in the internal cohort. **(E)** Representative IHC staining images of SCG2 between colorectal cancer and normal tissue. **(F)** Survival analysis of in-house cohort with RFS. **(G)** Univariate analysis of internal cohort with relapse-free survival (RFS) as the outcome event. **(H)** Multi-factor regression analysis of internal and external queues with relapse-free survival (RFS) as the outcome event. ns, *P >*0.05; ***P <0.001.

To further dissect the potential mechanisms of the above modules, we conducted the KEGG enrichment analysis and GO enrichment analysis of the 793 genes in the modules based on the R package “clusterProfiler”. The outcomes showed that 51 GO terms and 32 KEGG pathways were significantly enriched in module genes. Some of these GO terms (extracellular matrix organization, ameboid-type cell migration, type I interferon signaling pathway), as well as part of the KEGG pathways (Metabolism of xenobiotics by cytochrome P450, Drug metabolism-cytochrome P450, ECM-receptor interaction), is displayed through the bubble diagram (**Figure 3A**). Afterward, we evaluated the area under the curve (AUC) values of all modular genes in the three chemotherapy cohorts to better identify the genes that can determine the response to CRC chemotherapy. Genes with AUC >0.7 in each chemotherapy cohort seem as core genes, which consist of 20 candidate genes shown in **Figure 3B**. One-way Cox analysis of the above genes yielded four signatures (GILS2, MAOB, SCG2, ZHX2) that consistently act as risk factors in more than half of the cohorts with relapse-free survival (RFS). Besides, in a one-way Cox analysis of samples with overall survival (OS), these signatures were still more closely related to prognosis than other genes (**Figures 3C**, **S1C**). For validating the effects of the signatures on prognosis, the patients in RFS and OS cohorts were allocated to groups according to the high or low expression of the 20 genes using the cutoff values acquired with the “survminer” package and performed Kaplan-Meier survival analyses and log-rank tests (**Figures S1D**, **S2A**). Quantitative PCR results showed that only SCG2 (*P <*0.001) expression in the tumor was markedly higher than that in adjacent normal tissues (**Figure 3D**). Immunohistochemical staining for SCG2 in CRC specimens showed the same trend (**Figure 3E**). In addition, in the RFS survival analysis of the internal cohort, the group with high SCG2 (*P <*0.01) expression suggested a poor prognosis, and there was no statistically significant difference in MOAB (*P* =0.12), GLIS2 (*P* =0.18), and ZHX2 (*P* =0.095) (**Figure 3F**). However, in agreement with the outcomes from external cohorts, these four genes still showed good performance in predicting chemotherapy response (**Figure S2B**). Univariate and multifactorial Cox regression analysis indicated that SCG2 could be considered an independent predictor of prognosis (**Figures 3G, H**). Therefore, SCG2 may be a latent biological marker to predict chemotherapy response and prognosis.

### Exploring the Biological Characteristics of SCG2

**Figure 4:**
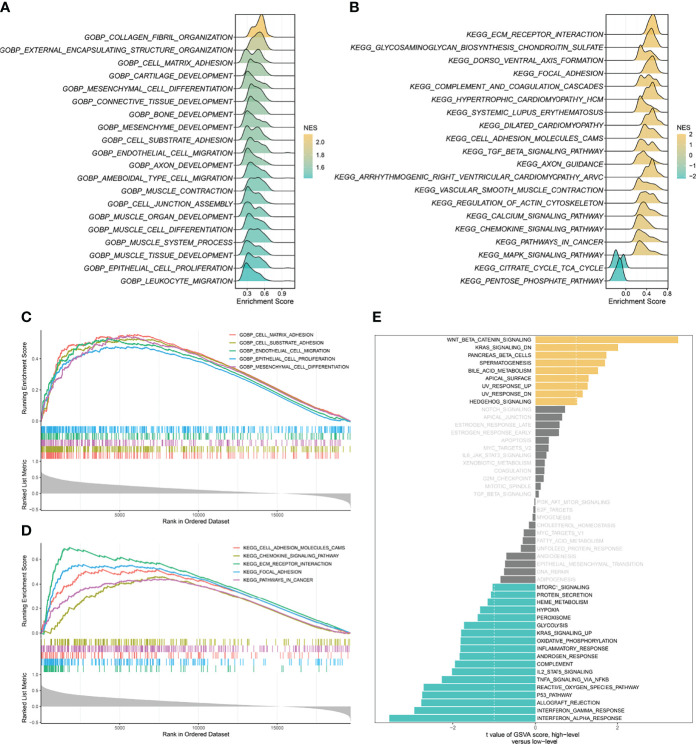
Gene set enrichment analysis (GSEA) and gene set variation analysis (GSVA) of SCG2. **(A)** Top 20 Gene Ontology (GO) terms with significant enrichment. **(B)** Top 20 Kyoto Encyclopedia of Genes and Genomes (KEGG) pathways with significant enrichment. **(C)** Five significantly enriched GO terms associated with SCG2. **(D)** Five Significantly enriched KEGG pathways associated with SCG2. **(E)** Differences in pathway activities scored by GSVA between high and low expression of SCG2.

Based on the correlation between SCG2 and other genes, the GSEA enrichment analysis revealed that SCG2 was involved in cancer invasion and growth signaling pathways. The GO terms shown in **Figure 4A** significantly enriched the genes highly related to SCG2, including cell-matrix adhesion, cell-substrate adhesion, mesenchymal cell differentiation, endothelial cell migration, and epithelial cell proliferation (**Figure 4C**). The KEGG pathways shown in **Figure 4B** include ECM receptor interaction, focal adhesion, cell adhesion molecules (CAMs), pathway in cancer, and chemokine signaling pathway (**Figure 4D**). The GSVA analysis of hallmark pathway gene signatures highlighted that, under the condition of differential expression between high and low, most changes focus on cancer development and progression mechanisms, such as KRAS signaling and the TP53 pathway (**Figure 4E**). Integrative analysis of biological pathways hints that SCG2 may be engaged in meditating tumor growth and invasion.

### Cell Culture and Functional Assay

**Figure 5:**
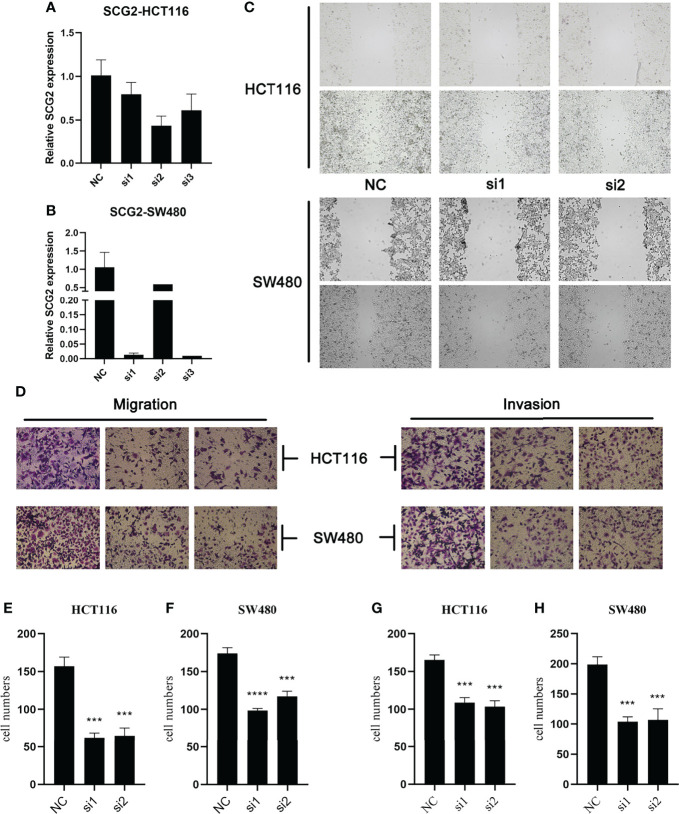
Effects of SCG2 on CRC cells migration and invasion. **(A, B)** Expression was significantly reduced after SCG2 knockdown in in HCT116 **(A)** and SW480 **(B)** cell lines. **(C)** Wound-healing assay to detect the migratory ability of CRC cells in the control group and SCG2 downregulation group. **(D)** Transwell assay to detect the migratory and invasive ability of CRC cells in the control group and SCG2 downregulation group. **(E–H)** SCG2 knockdown significantly inhibits migration and invasion behavior in HCT119 **(E, G)** and SW480 **(F, H)** cell lines. ***P <0.001; ****P <0.0001.

Specific shRNAs (sh-Control, sh-SCG2#1/2/3) and SCG2 plasmids were transfected into HCT116 and SW480 cancer cell lines, and the expression of the SCG2 gene was verified by qRT-PCR (**Figures 5A, B**). Then, wound-healing assays showed that depletion of SCG2 significantly inhibited the healing of scratched wounds (**Figure 5C**). In line with the above outcomes, Transwell assays confirmed that SCG2 snubbing suppressed CT116 and SW480 cells (**Figures 5D–H**). On the contrary, cancer cells with SCG2 overexpression demonstrated more aggressive migratory and invasive potential.

**Figure 6:**
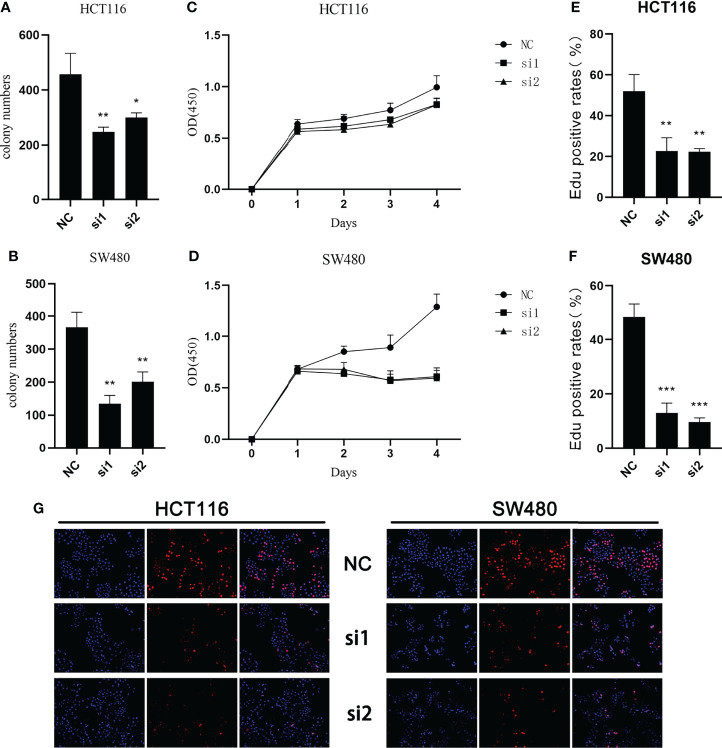
Effects of SCG2 on CRC cells proliferation. **(A, B)** SCG2 knockdown significantly reduced colony numbers of HCT116 **(A)** and SW480 **(B)** cell lines. **(C, D)** Reduced proliferative capacity of SCG2 knockdown HCT116 **(C)** and SW480 **(D)** cell lines in CCK8 array. **(E-G)** EdU assay **(G**, left: DAPI, middle: EdU, right: Merge**)** to detect the proliferative ability of CRC cells in the control group and SCG2 downregulation group. SCG2 knockdown significantly reduced the proliferative ability of HCT116 **(E)** and SW480 **(F)** cell lines. *P <0.05; **P <0.01; ***P <0.001.

In the study of cell proliferation, reduced SCG2 expression significantly suppressed the cell colony formation capacity in HCT116 and SW480 cells (**Figures 6A, B**). The CCK8 assay showed that significant differences in cell proliferation could be observed in HCT116 (*P <*0.05) and SW480 (*P <*0.05) cells with knockdown of SCG2 compared to control cells (**Figures 6C, D**). Further validation by EdU assay showed that knockdown of SCG2 in two cell lines markedly restrained cell proliferation, and the control group with SCG2 overexpression proliferated actively (**Figures 6E–G**). This result hints that overexpression of SCG2 is closely associated with CRC proliferation.

### Gene Mutation and Copy Number Variation Analysis

**Figure 7:**
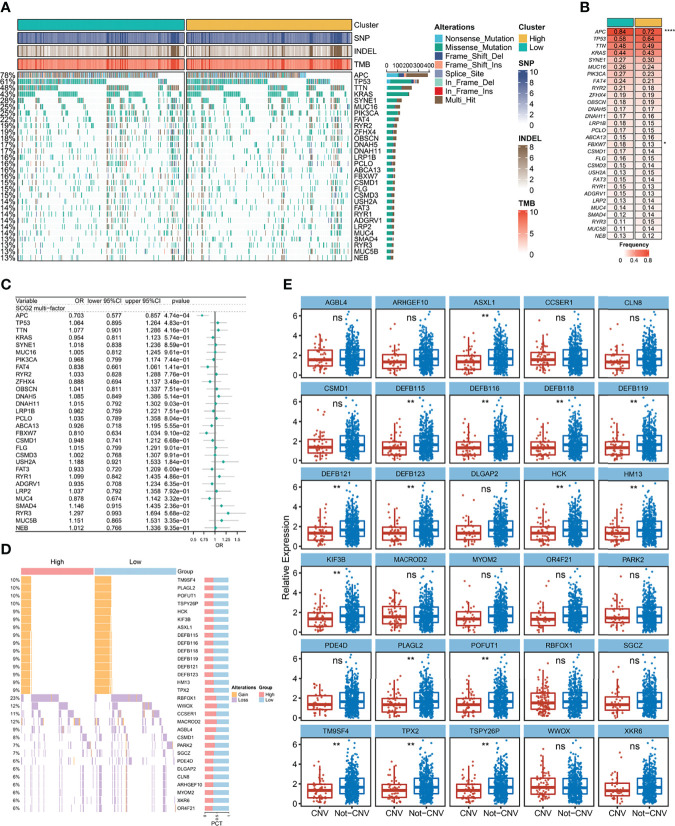
Gene mutation and copy number variation analysis. **(A)** Mutation landscape between high and low expression groups of SCG2, including a total of 30 frequently mutated genes (FMGs). **(B)** Analysis of mutational differences in FMGs between SCG2 expression subgroups. **(C)** A multivariate logistic regression analysis of FMGs, which incorporated SCG2 expression, TMB, age, gender, and stage. **(D)** Copy number variation landscape between high and low expression groups of SCG2. **(E)** Expression differences of SCG2 between variant and non-variant groups in top 15 deletion and amplification genes of copy number variation. *P <0.05; **P <0.01; ****P <0.0001; ns, *P* >0.05.

A total of 30 frequently mutated genes (FMGs) were identified in the CRC patients from TCGA, consisting APC (78%), TP53 (61%), TTN (48%), KRAS (43%), SYNE1 (28%), MUC16 (25%), PIK3CA (25%), FAT4 (22%), RYR2 (19%), ZFHX4 (19%), OBSCN (18%), DNAH5 (17%), DNAH11 (17%), LRP1B (16%), PCLO (16%), ABCA13 (16%), FBXW7 (16%), CSMD1 (15%), FLG (15%), CSMD3 (15%), USH2A (14%), FAT3 (14%), RYR1 (14%), ADGRV1 (14%), LRP2 (14%), MUC4 (14%), SMAD4 (13%), RYR3 (13%), MUC5B (13%), NEB (13%; **Figure 7A**). Despite the high mutation frequency of FMGs in CRC, there were no significant differences in TMB between SCG2-expressing subgroups and no statistically significant correlation analysis between TMB and SCG2 (**Figures S3A, B**). Similarly, neoantigen load, which is highly related to TMB, was not significantly associated with SCG2 expression (**Figure S3C**). The mutation frequencies of APC and ABCA13 were dramatically distinct between samples from the high and low SCG2 expression groups (**Figure 7B**). Nevertheless, only APC appeared significant expression differences of SCG2 between mutation and non-mutation groups in FMGs (**Figure S3D**). Univariate logistics regression with SCG2 expression as the independent variable indicated that SCG2 expression is an important factor influencing for APC mutation (**Figure S3E**). The above results were confirmed in multivariate logistics regression analysis covering variables of TMB, age, gender, stage, and SCG2 expression (**Figure 7C**). In the copy number variation analysis, the top 15 genes with the highest frequency of amplification and deletion, respectively, are shown in **Figure 7D**. Among them, the expression levels of SCG2 present a statistically significant difference between mutant and non-mutant subgroups of all amplified genes (**Figure 7E**). Univariate and multivariate logistic regression analysis revealed that SCG2 expression was an independent factor for all amplified genes mutation (**Figure S3G**). However, At the arm level, both the gain and loss loads were not significantly associated with SCG2 expression (**Figure S3F**).

### Immune Infiltration Associated With SCG2 Expression

**Figure 8:**
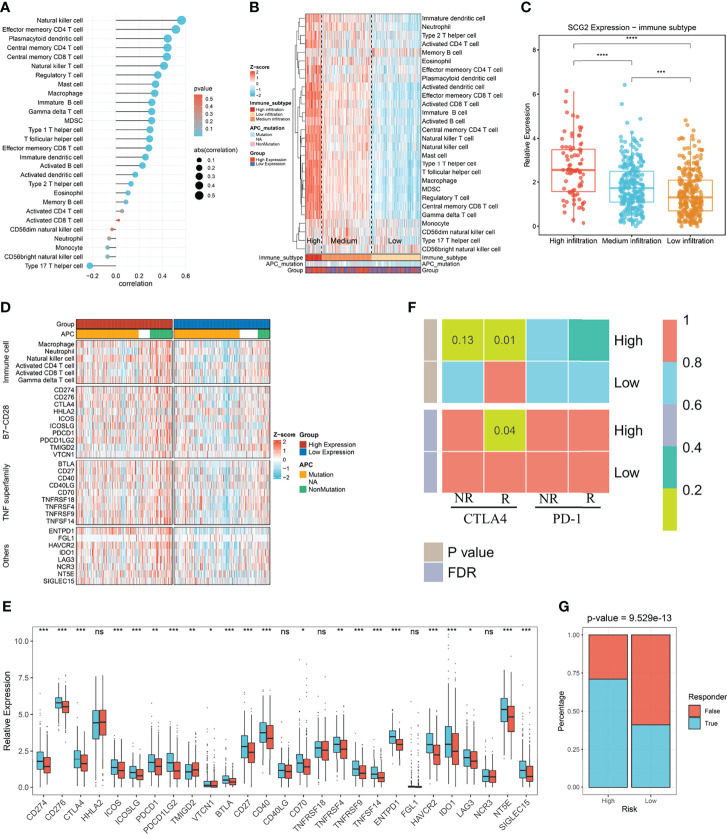
Immune infiltration associated with SCG2 expression and immunotherapy prognosis. **(A)** Correlation of immune cell infiltration score with SCG2 expression. **(B)** Distribution of immune cell infiltration and clinical features among the three subtypes obtained by hierarchical clustering. **(C)** Significant differences in SCG2 expression among the three immune subtypes. **(D)** Differential expression analysis of molecules representing immune characteristics in different SCG2 expression subgroups. **(E)** Differences in immune checkpoint expression between high and low SCG2 expression groups. **(F)** SubMap algorithm evaluated the expression similarity between the two SCG2 expression subgroups and the patients with a different immunotherapy response. **(G)** Distribution of immunotherapy responders predicted by TIDE algorithm between high and low SCG2 expression groups. *P <0.05; **P <0.01; ***P <0.001; ****P <0.0001; ns, *P* >0.05.

We utilized the ssGSEA approach to deconvolve the relative abundance of each immune cell type with transcriptome expression profiling data retrieved from the TCGA repository. Correlation analysis between SCG2 expression samples and 28 immune cell infiltration scores in CRC samples suggested that 22 cells were significantly associated with SCG2 (**Figure 8A**). According to the immune cell infiltration score, hierarchical clustering was used to stratify the samples into three groups: high, medium, and low. Samples with high SCG2 expression and APC mutation tend to be included in high immune infiltration (**Figure 8B**). There were likewise remarkable differences in SCG2 expression among samples from distinct immune infiltrate groups: SCG2 expression was highest in the high infiltration group, followed by the medium infiltration group, and lowest in the low infiltration group (**Figure 8C**).

Further study on the immune difference between high and low SCG2 groups showed the distinction of the immune landscape by ComplexHeatmap. The results showed that the immune cell score and the expression of immune-related molecular markers were significantly higher in the high SCG2 expression group than in the low expression group (**Figure 8D**). Immune checkpoints are generally overexpressed in the SCG2 high group. Molecular markers of immune checkpoints such as CD274, CD276, CTLA4, ICOS, ICOSLG, PDCD1, PDCD1LG2, TMIGD2, VTCN1, BTLA, CD27, CD40, CD70, TNFRSF9, TNFSF14, ENTPD1, HAVCR2, IDO1, LAG3, NT5E, and SIGLEC15 were significantly overexpressed in the SCG2 high group than the low group. Although the expression of HHLA2, CD40LG, TNFRSF18, FGL1, and NCR3 was not statistically different between the two groups, there was still a trend of overexpression in the high expression group (**Figure 8E**). Altogether, the molecular characterization of high SCG2 expression is of great significance for immunotherapy.

### Immunotherapy and Potential Drug Targets

We found significant expression similarity between patients with high SCG2 expression treated with anti-CTLA-4 by SubMap analysis, which revealed the same subtype sensitive to immunotherapy between independent cohorts (FDR <0.05; **Figure 8F**). The result follows the preliminary finding that immunotherapy may yield a better response in the high expression group. We performed the TIDE framework to estimate the immunotherapy response of each patient and encountered the ratio of responders to immunotherapy in high SCG2 expression was bigger than low SCG2 expression (high vs. low: 55% vs. 26%; P <0.001; **Figure 8G**). We operated CMap to explore prospect blends that might target pathways associated with the SCG2 gene. The results showed that 107 compounds were significantly enriched, and we selected the top 20 compounds based on specificity and obtained their mechanism of action by CMap Mode-of-action (MOA) analysis (**Figures S4A, B**).

## Discussion

Despite the inspiring advances of CRC chemotherapy so far, the response rate in patients continues to remain low, and the treatment benefits are uneven due to the development of chemoresistance. Such inter-individual differences may stem from the unique genetic and epigenetic make-up of each individual (23). Thus, there is a pressing demand for molecular identifications to guide clinical chemotherapy. The genomic transcriptome reflects tumor heterogeneity and promises personalized therapy (7). In our research, crucial modules associated with chemotherapy response yielded after WGCNA, and the survival analysis presented a valuable biomarker. Most of the pathways enriched in module genes are associated with extracellular matrix and drug metabolism, embodying the potential value of these genes in predicting response to chemotherapy. Multiple studies have demonstrated that elevated extracellular matrix in colorectal cancer tissues promotes aggressive tumor growth or increases drug resistance (24, 25), which further suggests a potential link between modular genes with drug response and tumor progression.

Comprehensive analysis of independent cohorts with OS or RFS of CRC indicated that the overall prognosis of patients in the SCG2 high group was higher than the low group, in agreement with the results of previously published studies (26). In the study of group 3 medulloblastoma, Eric et al. have verified these results (27). Functional pathways displaying cell adhesion and migration and immune cell infiltrations were prominent in the enrichment analysis of SCG2-associated genes, which further suggested the potential mining value of SCG2 in tumor development and immune microenvironment.

Adenomatous polyposis coli (APC) is a highly mutated oncogene in colorectal cancer. This gene’s mutation and inactivation are critical and premature events observed during CRC tumorigenesis (28). In our study, SCG2 expression was most closely associated with APC mutations as an independent influencing factor. APC is a critical negative regulator of the typical Wnt signaling pathway, which controls gastrointestinal cells’ coordinated proliferation and differentiation (29, 30). The previous studies have shown that genomic alterations of APC conduct to activation of β-catenin/T-cell factor transcriptional activity by rising nuclear β-catenin levels and attenuating CtBP-mediated repression of the repressor complex. The activation results in the upregulation of downstream target molecules, such as the cell cycle proteins D1 and Myc, which are essential motorists of tumor appearance due to their precise functions in cell proliferation, apoptosis, and cell cycle progression (28). Deactivation of APC is similarly thought to facilitate tumorigenesis *via* misplacement of cell-cell adhesion, possibly by regulating cell adhesion through the allocation of β-catenin and E-cadherin between the cytoplasm and cell membrane. APC mutations lack the binding domain of β-catenin, leading to diminished cell adhesion (31).

Interestingly, the GSEA enrichment results in this study suggest that SCG2-related pathways are also predominantly cell adhesion and migration. In addition, the Wnt/β-catenin signaling pathway was the highest-scoring related pathway in the GSVA study of SCG2 high and low expression groups. There may be a link between SCG2 and APC in the development of colorectal cancer, making SCG2 play an essential role in disease progression. Besides, this finding was also evidenced in the study of CNA. In previous investigations, TM9SF4, PLAGL2, and POFUT1, the most frequently amplified genes in our study, were strongly associated with the proliferation and metastasis of CRC (32–34). In addition, studies in CRC and breast cancers have shown that high TM9SF4 expression may promote chemoresistance and that knocking down the gene is not only for inhibiting tumor progression but also for enhancing chemotherapeutic sensitivity (33, 35). Therefore, we hypothesize that SCG2 can serve as a prognostic indicator and may also have the potential to predict drug response.

The prognosis of CRC is associated with the infiltration and activation of immune cells in the tumor microenvironment (36). Follow-up bioinformatics analysis showed that SCG2 was closely associated with immune progress, indicating the role of SCG2 in the immune microenvironment of CRC. In the current investigation, we observed that the SCG2 high expression group was featured by immune activation concomitant with immunosuppression; this characterization describes why immune activation was abundant in the SCG2 high expression group without impeding tumor progression. In general, tumor cells, immune cells, stromal cells, vascular endothelial cells, and their secreted factors and extracellular matrix components form a microenvironment that promotes tumor progression (37). In the early stages of tumor formation, these immune cells and associated stroma are recruited and activated, which creates an anti-tumor inflammatory environment that hinders tumor progression (13, 38). As tumors progress, immune cells infiltrating the tumor microenvironment, besides exerting anti-tumor effects, promote immune escape and tumor growth (13, 39). In recent decades, immunotherapy emerged as a hot spot in anti-tumor research and acted effectively on tumor treatment (40). Our study revealed that the SCG2 high expression group had an enriched immune response with significantly higher expression of CD28-B7, the co-stimulatory molecules that activate T cells, than the low expression group. The TNF superfamily, which is pivotal in the tumor microenvironment, is highly expressed in the high group. It suggests that the tumor microenvironment in the high SCG2-expressing group may incline to be immune hot compared to the low expression group. In addition, we uncovered that the SCG2 high group presented more elevated PD1, PDL1, and CTLA4 expression than the SCG2 low group, suggesting that the former is better conceivable to be in immunosuppressed circumstances, thereby suppressing the procedure of immune cells. Established on these conclusions, we scrutinized the relationship between SCG2 and immune checkpoints and the sensitivity of different expression groups of SCG2 to immunotherapeutic responses. Interestingly, TMB, as an essential biological indicator for predicting response to immunotherapy, was not significantly correlated with SCG2 expression. In CRC, high TMB is necessary but not sufficient to achieve long-lasting benefits, and above the critical threshold for hypermutation phenotypes, even CRC with very high TMB may not respond to treatment. This is different from lung cancer and melanoma, where the response is always positively correlated with TMB (41, 42). The further investigation exhibited a potent correlation between SCG2 and immune checkpoints and that a subgroup with high SCG2 expression was more sensitive to immunotherapy. Therefore, SCG2 has potential value for immunotherapy prediction. Combined with the fact that SCG2 obtained from the previous study can be considered a predictor of chemotherapy, it suggests that the combination of immune checkpoint therapy with chemotherapy in the group with high SCG2 expression may have unexpected benefits. Finally, CMap analysis provides more compounds that can be used as chemotherapy regimens for potential monotherapy or combination therapy in CRC treatment.

The biological heterogeneity of tumors has been plaguing the clinical choice of treatment options. While chemotherapy and immunotherapy serve as indispensable oncological therapeutic agents, selecting appropriate patients deserves more attention. However, there are currently no applicable clinical biomarkers to help doctors choose the appropriate treatment for CRC patients, improve the treatment effect and prolong survival, and reduce unnecessary side effects in other patients. Our study analyzed CRC chemotherapy data and validated results by *in vitro* experiments, providing a potential marker that can predict chemotherapy and immunotherapy responses, which offers the possibility to guide clinical treatment management. Moreover, treatment regimens with chemotherapy and immunotherapy are also essential in the clinic, suggesting a higher requirement for precision treatment in the future.

Despite these spottings, there remain some constraints. The sample data were accessed from the TCGA and GEO databases and did not provide precise details on the extent of surgical resection, an essential facet affecting overall survival. Data information stored in public databases is limited, and there is a lack of detailed transcriptome sequencing cohorts in this study to validate the obtained results, especially from the latest clinical samples. Therefore, more explicit clinical information should be provided for further analysis in the following study. In addition, we lacked sufficient clinical data to verify the predictive worth of SCG2 on the response to immunotherapy in CRC, which requires further efforts in our future studies. Finally, there is a lack of an internal cohort including chemotherapy and immunotherapy combination therapy to validate the conjecture of this study, which will be the emphasis of future research. While a comprehensive analysis of the multi-omics data from independent cohorts was conducted and some conclusions with clinical translation prospects were obtained, the molecular mechanism of SCG2 in the prognosis, chemotherapy, and immunotherapy of CRC should be further validated in more systematic molecular experiments.

## Conclusions

Our study suggests that SCG2 can be used as a new and effective indicator for predicting chemotherapy response, prognosis, and immune response in CRC patients, which means that SCG2 holds promise as a molecular marker to guide chemotherapy and immunotherapy in the clinical management of CRC. These results have important clinical implications and will contribute to the precise treatment of CRC patients.

## Data Availability Statement

The original contributions presented in the study are included in the article/**Supplementary Material**, further inquiries can be directed to the corresponding author/s.

## Ethics Statement

The studies involving human participants were reviewed and approved by Ethnics Committee of The First Affiliated Hospital of Zhengzhou University. The patients/participants provided their written informed consent to participate in this study.

## Author Contributions

ZQL and SYW designed the research route, and XWH performs conception, supervision, and funding acquisition. SYW executes the majority of data collation, analysis, experimental verification, and writing. HX, XYG, XFR, YYZ, RB, and JHD contributed to some data analysis and figures. ZQL and QD supported the completion of the experiments. YQR, LL, and CGG wrote the draft paper, and all the authors reviewed and modified the manuscript. All authors read and approved the final manuscript.

## Conflict of Interest

The authors declare that the research was conducted in the absence of any commercial or financial relationships that could be construed as a potential conflict of interest.

## Publisher’s Note

All claims expressed in this article are solely those of the authors and do not necessarily represent those of their affiliated organizations, or those of the publisher, the editors and the reviewers. Any product that may be evaluated in this article, or claim that may be made by its manufacturer, is not guaranteed or endorsed by the publisher.

## Supplementary Material

Supplementary Figure 1 Construction of co-expression network and prognostic analysis. **(A)** The topological overlap matrix (TOM) was transformed from the adjacency matrix. **(B)** The correlations between modules. **(C)** Univariate analysis with overall survival (OS) as the outcome event and the expression of the above 20 genes as independent variables. **(D)** Kaplan-Meier survival analysis of RFS cohorts from public portal.

Supplementary Figure 2 Prognostic analysis and efficacy of predicting chemotherapy response. **(A)** Kaplan-Meier survival analysis of OS cohorts from public portal. **(B)** The receiver operating characteristic curve assesses the efficacy of genes in predicting chemotherapy response in external and internal cohorts. **(C)** Differential expression of genes between chemotherapy-responsive and non-responsive groups in an internal cohort.

Supplementary Figure 3 Gene Mutation and Copy Number Variation Analysis. **(A)** Correlation analysis of TMB and SCG2 expression. **(B)** Differential analysis of the number of deletion and amplification mutations and TMB between high and low SCG2 expression groups. **(C)** Correlation analysis of neoantigen load and SCG2 expression. **(D)** Expression differences of SCG2 between mutation and non- mutation groups in top 30 FMGs. **(E)** A univariate logistic regression analysis of FMGs, which incorporated SCG2 expression as independent variable. **(F)** The association between the gain and loss loads with SCG2 expression at the arm level. **(G)** Univariate and multivariate logistic regression analysis revealed that SCG2 expression was an independent factor for all amplified genes mutation.

Supplementary Figure 4 Drug prediction. **(A)** CMap algorithm predicts drugs that may target SCG2 and ranks them by their specificity. **(B)** Mechanisms associated with the above drugs.

## References

[1] Sung H Ferlay J Siegel RL Laversanne M Soerjomataram I Jemal A. Global Cancer Statistics 2020: GLOBOCAN Estimates of Incidence and Mortality Worldwide for 36 Cancers in 185 Countries. CA Cancer J Clin (2021) 71(3):209–49. doi: 10.3322/caac.21660 33538338

[2] Dekker E Tanis PJ Vleugels JLA Kasi PM Wallace MB. Colorectal Cancer. Lancet (2019) 394(10207):1467–80. doi: 10.1016/s0140-6736(19)32319-0 31631858

[3] Doubeni CA Corley DA Quinn VP Jensen CD Zauber AG Goodman M. Effectiveness of Screening Colonoscopy in Reducing the Risk of Death From Right and Left Colon Cancer: A Large Community-Based Study. Gut (2018) 67(2):291–U53. doi: 10.1136/gutjnl-2016-312712 27733426 PMC5868294

[4] Levin TR Corley DA Jensen CD Schottinger JE Quinn VP Zauber AG. Effects of Organized Colorectal Cancer Screening on Cancer Incidence and Mortality in a Large Community-Based Population. Gastroenterology (2018) 155(5):1383–+. doi: 10.1053/j.gastro.2018.07.017 PMC6240353 30031768

[5] Allen WL Johnston PG. Role of Genomic Markers in Colorectal Cancer Treatment. J Clin Oncol (2005) 23(20):4545–52. doi: 10.1200/jco.2005.19.752 16002846

[6] Dienstmann R Salazar R Tabernero J. Personalizing Colon Cancer Adjuvant Therapy: Selecting Optimal Treatments for Individual Patients. J Clin Oncol (2015) 33(16):1787–+. doi: 10.1200/jco.2014.60.0213 25918287

[7] Linnekamp JF Wang X Medema JP Vermeulen L. Colorectal Cancer Heterogeneity and Targeted Therapy: A Case for Molecular Disease Subtypes. Cancer Res (2015) 75(2):245–49. doi: 10.1158/0008-5472.Can-14-2240 25593032

[8] Iveson TJ Sobrero AF Yoshino T Souglakos I Ou FS Meyers JP. Duration of Adjuvant Doublet Chemotherapy (3 or 6 Months) in Patients With High-Risk Stage II Colorectal Cancer. J Clin Oncol (2021) 39(15):1691–91. doi: 10.1200/jco.21.00954 PMC8078416 33439695

[9] Shi Q Paul J Grothey A. Duration of Adjuvant Chemotherapy for Stage III Colon Cancer REPLY. N Engl J Med (2018) 379(4):396–97. doi: 10.1056/NEJMc1805498 30044940

[10] Galon J Bruni D. Approaches to Treat Immune Hot, Altered and Cold Tumours With Combination Immunotherapies. Nat Rev Drug Discovery (2019) 18(3):197–218. doi: 10.1038/s41573-018-0007-y 30610226

[11] Ganesh K Stadler ZK Cercek A Mendelsohn RB Shia J Segal NH. Immunotherapy in Colorectal Cancer: Rationale, Challenges and Potential. Nat Rev Gastroenterol Hepatol (2019) 16(6):361–75. doi: 10.1038/s41575-019-0126-x PMC7295073 30886395

[12] Kather JN Halama N Jaeger D. Genomics and Emerging Biomarkers for Immunotherapy of Colorectal Cancer. Semin Cancer Biol (2018) 52:189–97. doi: 10.1016/j.semcancer.2018.02.010 29501787

[13] O'Donnell JS Teng MWL Smyth MJ. Cancer Immunoediting and Resistance to T Cell-Based Immunotherapy. Nat Rev Clin Oncol (2019) 16(3):151–67. doi: 10.1038/s41571-018-0142-8 30523282

[14] Rejhová A Opattová A Čumová A Slíva D Vodička P. Natural Compounds and Combination Therapy in Colorectal Cancer Treatment. Eur J Med Chem (2018) 144:582–94. doi: 10.1016/j.ejmech.2017.12.039 29289883

[15] Liu Z Guo C Dang Q Wang L Liu L Weng S. Integrative Analysis From Multi-Center Studies Identities a Consensus Machine Learning-Derived lncRNA Signature for Stage II/III Colorectal Cancer. EBioMedicine (2022) 75:103750. doi: 10.1016/j.ebiom.2021.103750 34922323 PMC8686027

[16] Langfelder P Horvath S. WGCNA: An R Package for Weighted Correlation Network Analysis. BMC Bioinf (2008) 9:13. doi: 10.1186/1471-2105-9-559 PMC2631488 19114008

[17] Langfelder P Horvath S. Fast R Functions for Robust Correlations and Hierarchical Clustering. J Stat Software (2012) 46(11):1–17. doi: 10.18637/jss.v046.i11 PMC3465711 23050260

[18] Lambrechts D Wauters E Boeckx B Aibar S Nittner D Burton O. Phenotype Molding of Stromal Cells in the Lung Tumor Microenvironment. Nat Med (2018) 24(8):1277–+. doi: 10.1038/s41591-018-0096-5 29988129

[19] Charoentong P Finotello F Angelova M Mayer C Efremova M Rieder D. Pan-Cancer Immunogenomic Analyses Reveal Genotype-Immunophenotype Relationships and Predictors of Response to Checkpoint Blockade. Cell Rep (2017) 18(1):248–62. doi: 10.1016/j.celrep.2016.12.019 28052254

[20] Jiang P Gu SQ Pan D Fu JX Sahu A Hu XH. Signatures of T Cell Dysfunction and Exclusion Predict Cancer Immunotherapy Response. Nat Med (2018) 24(10):1550–+. doi: 10.1038/s41591-018-0136-1 PMC6487502 30127393

[21] Hoshida Y Brunet JP Tamayo P Golub TR Mesirov JP. Subclass Mapping: Identifying Common Subtypes in Independent Disease Data Sets. PloS One (2007) 2(11):e1195. doi: 10.1371/journal.pone.0001195 18030330 PMC2065909

[22] Malta TM Sokolov A Gentles AJ Burzykowski T Poisson L Weinstein JN. Machine Learning Identifies Stemness Features Associated With Oncogenic Dedifferentiation. Cell (2018) 173(2):338–+. doi: 10.1016/j.cell.2018.03.034 PMC5902191 29625051

[23] Vodenkova S Buchler T Cervena K Veskrnova V Vodicka P Vymetalkova V. 5-Fluorouracil and Other Fluoropyrimidines in Colorectal Cancer: Past, Present and Future. Pharmacol Ther (2020) 206:19. doi: 10.1016/j.pharmthera.2019.107447 31756363

[24] Kasashima H Duran A Martinez-Ordonez A Nakanishi Y Kinoshita H Linares JF. Stromal SOX2 Upregulation Promotes Tumorigenesis Through the Generation of a SFRP1/2-Expressing Cancer-Associated Fibroblast Population. Dev Cell (2021) 56(1):95–+. doi: 10.1016/j.devcel.2020.10.014 PMC7856011 33207226

[25] Unterleuthner D Neuhold P Schwarz K Janker L Neuditschko B Nivarthi H. Cancer-Associated Fibroblast-Derived WNT2 Increases Tumor Angiogenesis in Colon Cancer. Angiogenesis (2020) 23(2):159–77. doi: 10.1007/s10456-019-09688-8 PMC7160098 31667643

[26] Sun GW Li YL Peng YJ Lu DP Zhang FQ Cui XY. Identification of a Five-Gene Signature With Prognostic Value in Colorectal Cancer. J Cell Physiol (2019) 234(4):3829–36. doi: 10.1002/jcp.27154 30132881

[27] Thompson EM Keir ST Venkatraman T Lascola C Yeom KW Nixon AB. The Role of Angiogenesis in Group 3 Medulloblastoma Pathogenesis and Survival. Neuro-Oncology (2017) 19(9):1217–27. doi: 10.1093/neuonc/nox033 PMC5570262 28379574

[28] Zhang L Shay JW. Multiple Roles of APC and its Therapeutic Implications in Colorectal Cancer. JNCI J Natl Cancer Inst (2017) 109(8):10. doi: 10.1093/jnci/djw332 PMC5963831 28423402

[29] Klaus A Birchmeier W. Wnt Signalling and its Impact on Development and Cancer. Nat Rev Cancer (2008) 8(5):387–98. doi: 10.1038/nrc2389 18432252

[30] Koni M Pinnaro V Brizzi MF. The Wnt Signalling Pathway: A Tailored Target in Cancer. Int J Mol Sci (2020) 21(20):26. doi: 10.3390/ijms21207697 PMC7589708 33080952

[31] Bienz M Hamada F. Adenomatous Polyposis Coli Proteins and Cell Adhesion. Curr Opin Cell Biol (2004) 16(5):528–35. doi: 10.1016/j.ceb.2004.08.001 15363803

[32] Li DJ Lin CW Li NP Du YH Yang CX Bai Y. PLAGL2 and POFUT1 are Regulated by an Evolutionarily Conserved Bidirectional Promoter and are Collaboratively Involved in Colorectal Cancer by Maintaining Stemness. EBioMedicine (2019) 45:124–38. doi: 10.1016/j.ebiom.2019.06.051 PMC6642334 31279780

[33] Lozupone F Borghi M Marzoli F Azzarito T Matarrese P Iessi E. TM9SF4 is a Novel V-ATPase-Interacting Protein That Modulates Tumor pH Alterations Associated With Drug Resistance and Invasiveness of Colon Cancer Cells. Oncogene (2015) 34(40):5163–74. doi: 10.1038/onc.2014.437 25659576

[34] Wu L Zhou ZL Han SB Chen JH Liu ZY Zhang XD. PLAGL2 Promotes Epithelial-Mesenchymal Transition and Mediates Colorectal Cancer Metastasis *via* Beta-Catenin-Dependent Regulation of ZEB1. Br J Cancer (2020) 122(4):578–89. doi: 10.1038/s41416-019-0679-z PMC7028997 31827238

[35] Zhu YF Xie MX Meng ZY Leung LK Chan FL Hu X. Knockdown of TM9SF4 Boosts ER Stress to Trigger Cell Death of Chemoresistant Breast Cancer Cells. Oncogene (2019) 38(29):5778–91. doi: 10.1038/s41388-019-0846-y 31249383

[36] Liu Z Guo C Dang Q Wang L Liu L Weng S. Integrative analysis from multi-center studies identities a consensus machine learning-derived lncRNA signature for stage II/III colorectal cancer. EBioMedicine (2021) 75:103750. doi: 10.1016/j.ebiom.2021.103750 34922323 PMC8686027

[37] Bejarano L Jordao MJC Joyce JA. Therapeutic Targeting of the Tumor Microenvironment. Cancer Discovery (2021) 11(4):933–59. doi: 10.1158/2159-8290.Cd-20-1808 33811125

[38] Ozga AJ Chow MT Luster AD. Chemokines and the Immune Response to Cancer. Immunity (2021) 54(5):859–74. doi: 10.1016/j.immuni.2021.01.012 PMC8434759 33838745

[39] Del Alcazar CRG Aleckovic M Polyak K. Immune Escape During Breast Tumor Progression. Cancer Immunol Res (2020) 8(4):422–27. doi: 10.1158/2326-6066.Cir-19-0786 PMC7138346 32238387

[40] Del Paggio JC. Immunotherapy Cancer Immunotherapy and the Value of Cure. Nat Rev Clin Oncol (2018) 15(5):268–+. doi: 10.1038/nrclinonc.2018.27 29459643

[41] Bortolomeazzi M Keddar MR Montorsi L Acha-Sagredo A Benedetti L Temelkovski D. Immunogenomics of Colorectal Cancer Response to Checkpoint Blockade: Analysis of the KEYNOTE 177 Trial and Validation Cohorts. Gastroenterology (2021) 161(4):1179–93. doi: 10.1053/j.gastro.2021.06.064 PMC8527923 34197832

[42] Wood MA Weeder BR David JK Nellore A Thompson RF. Burden of Tumor Mutations, Neoepitopes, and Other Variants are Weak Predictors of Cancer Immunotherapy Response and Overall Survival. Genome Med (2020) 12(1):33. doi: 10.1186/s13073-020-00729-2 32228719 PMC7106909

