# Supplementary figures and images for: SCG2: A Prognostic Marker That Pinpoints Chemotherapy and Immunotherapy in Colorectal Cancer
Source: Front Immunol. 2022 Jul 1;13:873871. doi: 10.3389/fimmu.2022.873871 (PMC9283651; doi:10.3389/fimmu.2022.873871)

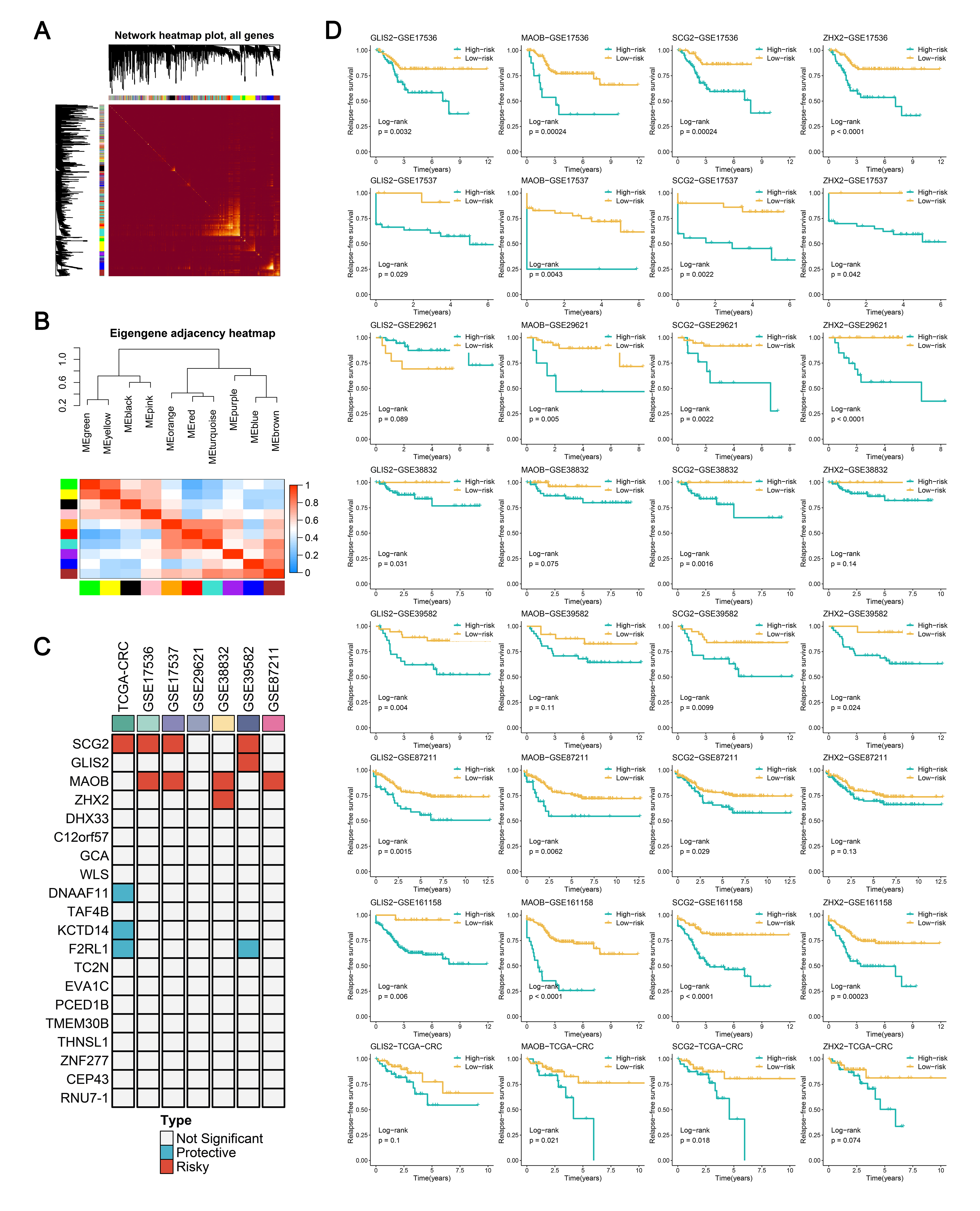

Supplement: Supplementary Figure 1 — Construction of co-expression network and prognostic analysis. (A) The topological overlap matrix (TOM) was transformed from the adjacency matrix. (B) The correlations between modules. (C) Univariate analysis with overall survival (OS) as the outcome event and the expression of the above 20 genes as independent variables. (D) Kaplan-Meier survival analysis of RFS cohorts from public portal. [file Image_1.jpeg]

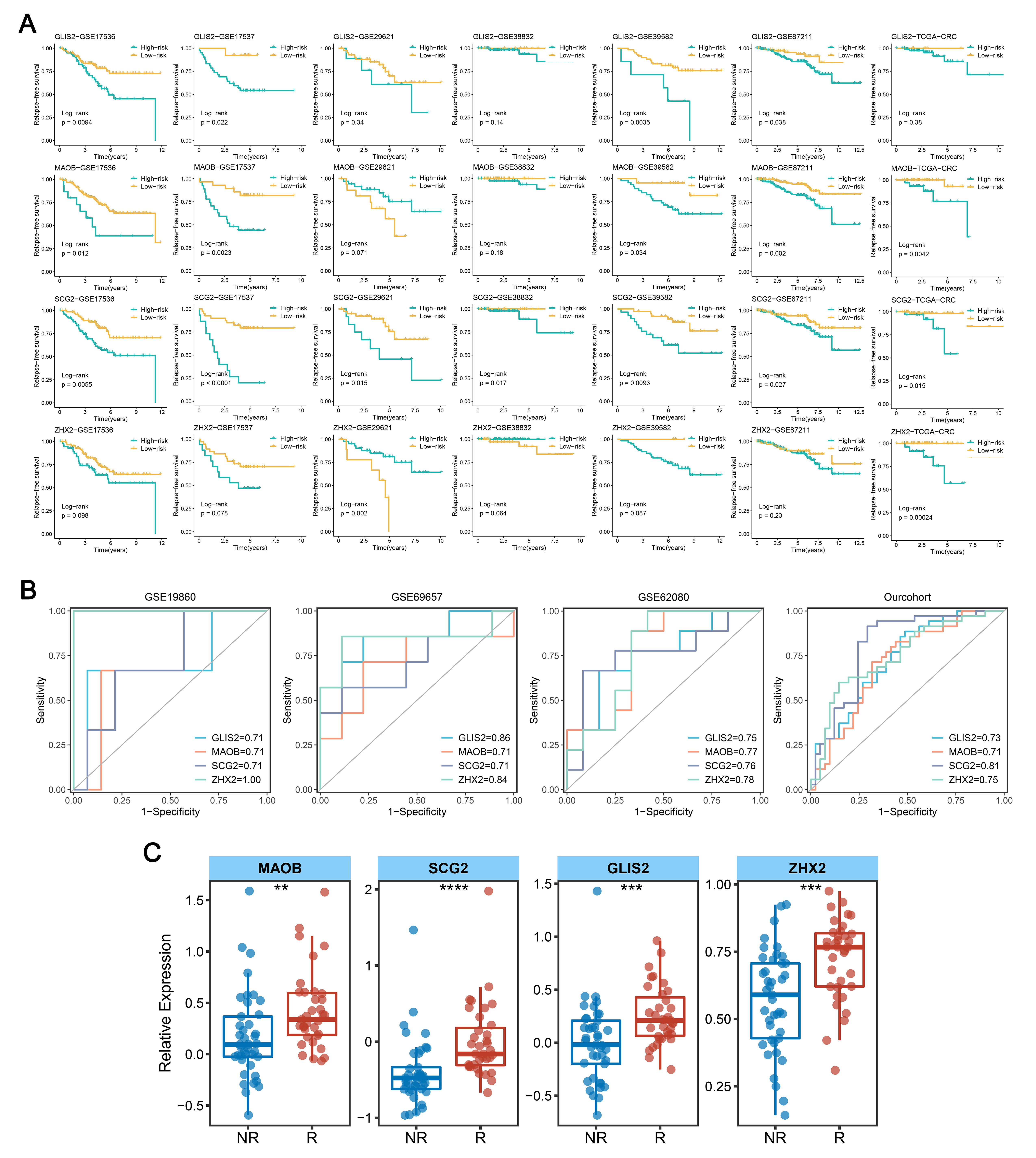

Supplement: Supplementary Figure 2 — Prognostic analysis and efficacy of predicting chemotherapy response. (A) Kaplan-Meier survival analysis of OS cohorts from public portal. (B) The receiver operating characteristic curve assesses the efficacy of genes in predicting chemotherapy response in external and internal cohorts. (C) Differential expression of genes between chemotherapy-responsive and non-responsive groups in an internal cohort. [file Image_2.jpeg]

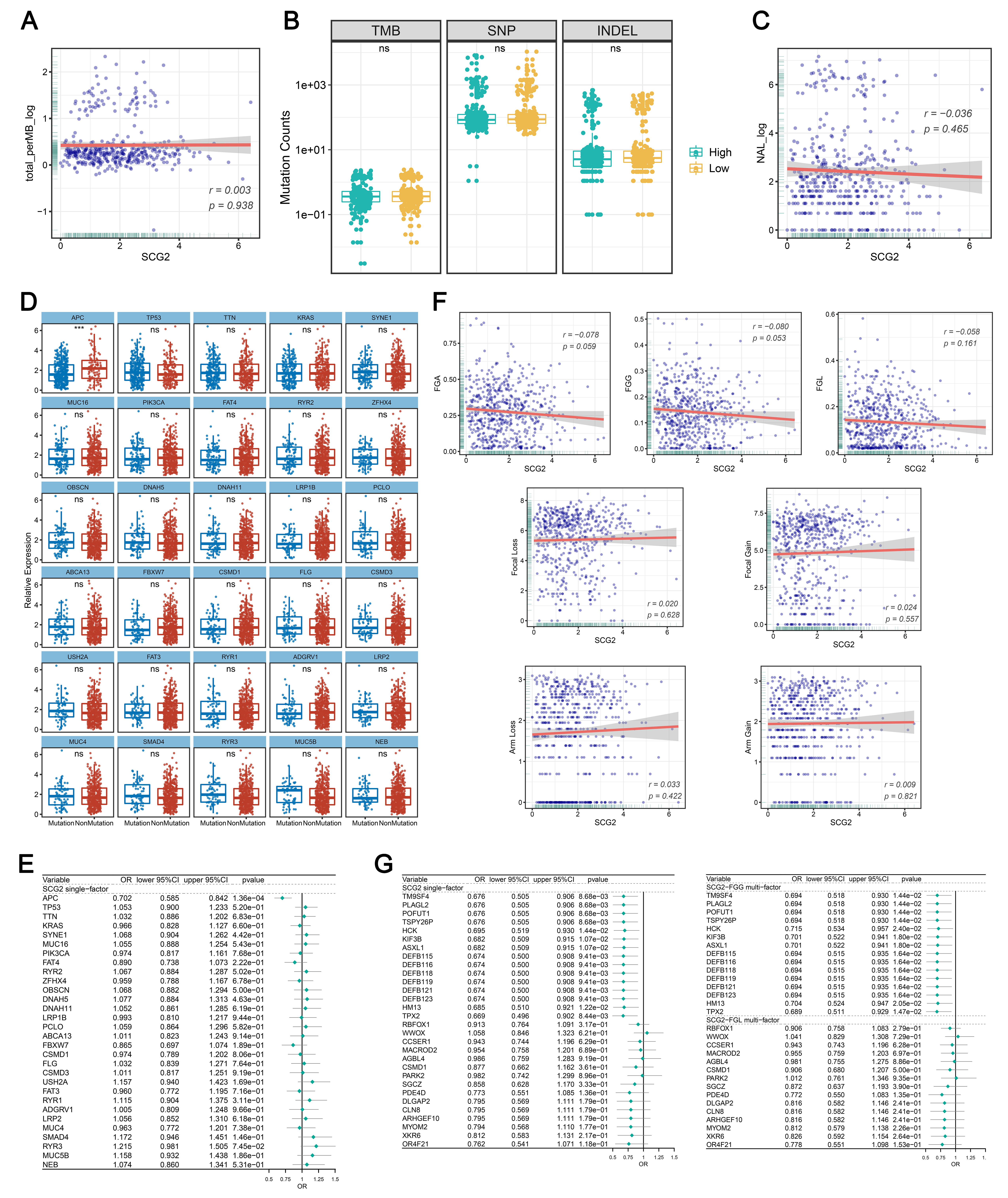

Supplement: Supplementary Figure 3 — Gene Mutation and Copy Number Variation Analysis. (A) Correlation analysis of TMB and SCG2 expression. (B) Differential analysis of the number of deletion and amplification mutations and TMB between high and low SCG2 expression groups. (C) Correlation analysis of neoantigen load and SCG2 expression. (D) Expression differences of SCG2 between mutation and non- mutation groups in top 30 FMGs. (E) A univariate logistic regression analysis of FMGs, which incorporated SCG2 expression as independent variable. (F) The association between the gain and loss loads with SCG2 expression at the arm level. (G) Univariate and multivariate logistic regression analysis revealed that SCG2 expression was an independent factor for all amplified genes mutation. [file Image_3.jpeg]

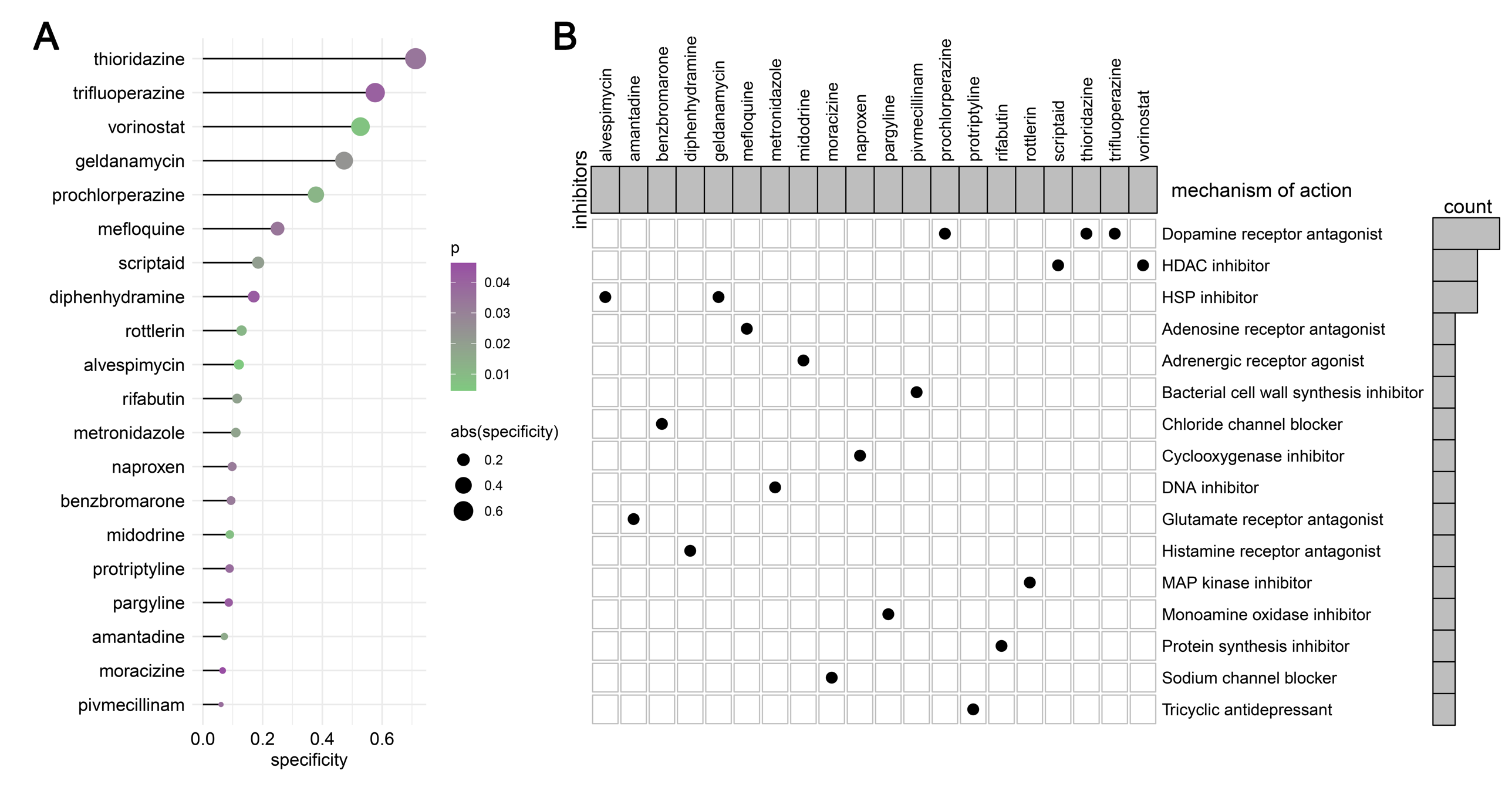

Supplement: Supplementary Figure 4 — Drug prediction. (A) CMap algorithm predicts drugs that may target SCG2 and ranks them by their specificity. (B) Mechanisms associated with the above drugs. [file Image_4.jpeg]
